# Preoperative Coagulation Markers and Clinical Predictors of Transfusion Requirement in Patients Undergoing Total Knee or Hip Arthroplasty: A Single-Center Retrospective Study

**DOI:** 10.3390/medsci13030135

**Published:** 2025-08-15

**Authors:** Wojciech Konarski

**Affiliations:** Medical Rehabilitation Center, Sobieskiego 47D, 05-120 Legionowo, Poland; wkonarski@poczta.onet.pl; Tel.: +48-502110863

**Keywords:** transfusion requirement, total hip arthroplasty, total knee arthroplasty, preoperative assessment, coagulation markers, hemoglobin

## Abstract

**Background/Objectives:** Total knee arthroplasty (TKA) and total hip arthroplasty (THA) are widely performed procedures often associated with significant blood loss, leading to the need for allogeneic blood transfusion. Transfusions carry inherent risks and increase healthcare costs, making the identification of transfusion predictors crucial. This study aimed to assess preoperative predictors associated with transfusion requirement in patients undergoing THA or TKA. **Methods:** This single-center, retrospective analysis included 742 patients who underwent primary TKA or THA between 2016 and 2023. Preoperative variables such as hemoglobin, red blood cell count (RBC), INR, APTT, and use of tranexamic acid (TXA) were collected. Univariable and multivariable logistic regression analyses were conducted to identify independent predictors of transfusion. **Results:** Transfusions were required in 12.0% of patients. Multivariable analysis revealed that lower preoperative HGB and RBC levels, absence of TXA use, higher INR, and undergoing THA (versus TKA) were independently associated with increased transfusion risk. INR was not significant in univariable analysis but reached significance in the adjusted model. The final multivariable model demonstrated good predictive performance, with an area under the ROC curve (AUC) of 0.79. **Conclusions:** Lower hemoglobin and RBC levels, elevated INR, absence of TXA use, and THA surgery were independent predictors of transfusion. These findings may guide the use of routine preoperative hematologic and coagulation assessments to guide perioperative management and reduce transfusion rates in joint arthroplasty.

## 1. Introduction

Total knee arthroplasty (TKA) and total hip arthroplasty (THA) are among the most commonly performed orthopedic procedures worldwide [1,2,3,4], also in Poland [5]. One of the most frequent indications for TKA is osteochondritis dissecans, while avascular necrosis (AVN) remains a leading cause for THA [6,7,8]. Although both interventions offer substantial improvements in pain, mobility, and quality of life, they are associated with considerable intraoperative and postoperative blood loss, often necessitating allogeneic blood transfusion [9,10,11]. The use of transfusions, while sometimes essential, carries the risk of complications such as transfusion reactions, infections, and prolonged hospital stay, and can increase overall healthcare costs [12,13]. Consequently, identifying preoperative factors that predict transfusion requirement is of clinical importance. Hemoglobin (HGB) and red blood cell (RBC) counts are well-established hematologic markers associated with perioperative blood management. However, the role of coagulation parameters such as the international normalized ratio (INR) and activated partial thromboplastin time (APTT) remains less clearly defined in this context. Additionally, the use of antifibrinolytic agents, such as tranexamic acid (TXA), has become a common strategy to minimize perioperative bleeding and transfusion rates, yet its impact in different patient subgroups continues to be evaluated [14,15,16,17,18,19].

Some studies have attempted to identify predictors of transfusion after joint arthroplasty, but many have focused primarily on demographic and surgical factors, with limited data on the contribution of baseline coagulation profiles. Furthermore, findings have often been inconsistent due to variations in transfusion thresholds, surgical techniques, perioperative protocols, and relatively small cohorts of patients [17,18,20].

In line with broader recommendations for perioperative optimization, recent systematic reviews have emphasized the importance of individualized coagulation assessment and structured hemostasis management, particularly in patients at elevated bleeding risk [21]. Although these insights were drawn primarily from hemophilic populations, the underlying principles such as careful evaluation of coagulation parameters and multidisciplinary planning remain relevant in the context of elective arthroplasty and support the rationale for integrating coagulation markers into transfusion risk models [21].

Real-world data evaluating the relationship between preoperative laboratory parameters and transfusion outcomes are still limited, particularly in mixed TKA/THA cohorts. Furthermore, it remains unclear to what extent standard coagulation markers, in combination with other clinical variables, can inform transfusion risk models and perioperative decision-making.

Therefore, the aim of this retrospective study was to analyze preoperative parameters, including standard coagulation tests, to identify predictors of transfusion requirement in patients undergoing TKA or THA. We also evaluated the role of TXA use in modulating transfusion risk in this population.

## 2. Materials and Methods

### 2.1. Study Design

This retrospective, single-center study was conducted at the Department of Trauma and Orthopedic Surgery, Voivodeship Specialist Hospital in Ciechanów, Poland. Adult patients who underwent primary THA due to AVN or TKA due to OCD, both classified as stage III or IV (Ficat and Guhl classifications, respectively), between 2016 and 2023 were eligible. Patient selection was based on a review of surgical logs and hospital records. All patients had provided written consent for the procedure. Ethical approval was waived due to the retrospective nature of the analysis, which was conducted in accordance with institutional guidelines.

We collected demographic characteristics (age, sex, BMI), pre- and postoperative laboratory values (HGB, RBC, INR, APTT), and transfusion requirements. TXA use prior to surgery was also recorded.

### 2.2. Surgical Procedures and Perioperative Management

All TKAs were performed using a medial parapatellar approach, while THAs were carried out using a posterior–lateral approach. Thromboprophylaxis was applied across all patients, consisting of a single subcutaneous dose of enoxaparin (0.4 mL) administered at least 12 h prior to surgery to minimize the risk of thromboembolic events.

Postoperative HGB levels were routinely monitored to estimate blood loss and guide transfusion decisions. Patients received transfusion when HGB dropped below 8.9 g/dL or decreased by more than 30% from baseline, particularly in the presence of clinical symptoms such as pallor, fatigue, or drowsiness; this reflects the standard institutional protocol used at the study center.

Postoperative laboratory evaluations were performed according to institutional protocol. Standard testing was typically conducted 24 h after the procedure. However, in cases where the patient’s condition raised clinical concern manifested by signs such as pallor, weakness, or visible anemia, tests could be performed as early as 12 h postoperatively to enable timely intervention.

### 2.3. Statistical Analysis

Baseline characteristics of the study population were summarized using descriptive statistics. Continuous variables were reported as mean ± standard deviation (SD). Categorical variables were expressed as counts and percentages.

Comparisons between patients who required blood transfusion and those who did not were performed using Student’s *t*-test or Mann–Whitney U test for continuous variables, and the chi-square test or Fisher’s exact test for categorical variables. A *p*-value < 0.05 was considered statistically significant.

To assess the individual association between each clinical variable and the need for blood transfusion, univariable logistic regression models were fitted. Each predictor was entered into a separate model with blood transfusion (yes/no) as the binary outcome. Odds ratios (ORs) with corresponding 95% confidence intervals (CIs) and *p*-values were reported.

To identify factors associated with the need for blood transfusion, a multivariate logistic regression analysis was performed. The outcome variable was binary (transfusion need: yes/no). Predictor variables included age, sex, BMI, use of tranexamic acid prior to surgery, HGB, RBC, INR, and APTT measured preoperatively. Patients with missing data in any of these variables were excluded from the regression analysis. Categorical variables were encoded using one-hot encoding (with reference categories dropped). Variables were standardized before model fitting. ORs with 95% CIs and *p*-values were reported. Statistical significance was set at *p* < 0.05. Patients with missing data for any of the variables included in the multivariable regression model (hemoglobin, RBC count, INR, APTT, TXA use, sex, age, BMI, and surgery type) were excluded from the analysis.

To evaluate the discriminatory performance of the multivariable logistic regression model, we calculated the area under the receiver operating characteristic (ROC) curve (AUC). Additionally, to determine the optimal probability threshold for classifying patients as high risk for transfusion, we used Youden’s index, defined as sensitivity + specificity–1.

The calculations were performed using Statistica software (version 14.0, TIBCO Software Inc., Palo Alto, CA, USA) and Python 3.11 with the scikit-learn 1.4.2 library.

## 3. Results

### 3.1. Baseline Characteristics

Table 1 presents the baseline characteristics of the study population (*n* = 742). The mean age of patients was 65.8 ± 9.9 years, and the average BMI was 30.1 ± 4.6 kg/m^2^. Most of the patients were women (61.1%). A slightly higher proportion of patients underwent THA (54.5%) compared to TKA (45.5%). The mean preoperative hemoglobin concentration was 13.9 ± 4.2 g/dL, and the mean RBC count was 4.5 ± 0.8 million/μL. The use of tranexamic acid before surgery was reported in 46.9% of patients. The overall transfusion rate was 12.0%.

### 3.2. Comparison of Patients with and Without Transfusion

Table 2 compares the demographic and clinical characteristics of patients who required blood transfusion (*n* = 89) with those who did not (*n* = 653). Transfused patients were significantly older (69.1 vs. 65.4 years, *p* = 0.0040) and had lower BMI (28.9 vs. 30.2 kg/m^2^, *p* = 0.0169). Women were more frequently transfused (75.3% vs. 59.2%, *p* = 0.0050). Preoperative HGB and RBC levels were significantly lower in the transfused group (HGB: 12.4 vs. 14.2 g/dL, *p* < 0.0001; RBC: 4.3 vs. 4.6 million/μL, *p* = 0.0221). A significantly lower proportion of these patients received TXA (18.0% vs. 50.8%, *p* < 0.0001). There were no significant differences in surgery type, INR, or APTT between groups.

### 3.3. Univariable Predictors of Transfusion Requirement

As shown in Table 3, the univariable analysis included 595 patients. Higher age and lower BMI were significantly associated with increased transfusion risk. Lower preoperative HGB and RBC values were strong predictors of transfusion, while male sex and TKA (vs. THA) were linked to lower risk. The strongest protective factor was TXA use (OR 0.22; *p* < 0.0001). INR and APTT were not significant.

### 3.4. Multivariable Predictors of Transfusion Requirement

**Figure 1:**
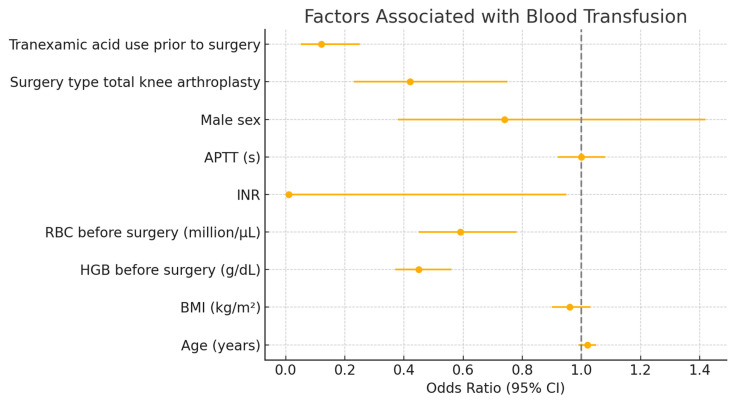
Factors independently associated with blood transfusion in the multivariate analysis. Abbreviations: APTT—activated partial thromboplastin time; BMI—body mass index; HGB—hemoglobin; INR—international normalized ratio; RBC—red blood cell count.

A total 147 patients (19.8%) were excluded from the multivariable analysis due to missing values. In the multivariable analysis (*n* = 595), lower preoperative HGB and RBC levels, lack of tranexamic acid use, and undergoing hip rather than knee arthroplasty remained significantly associated with increased transfusion risk. The INR variable also reached statistical significance (*p* = 0.0475). Age, sex, BMI, and APTT were not significant in the adjusted model (Table 4, Figure 1).

**Figure 2:**
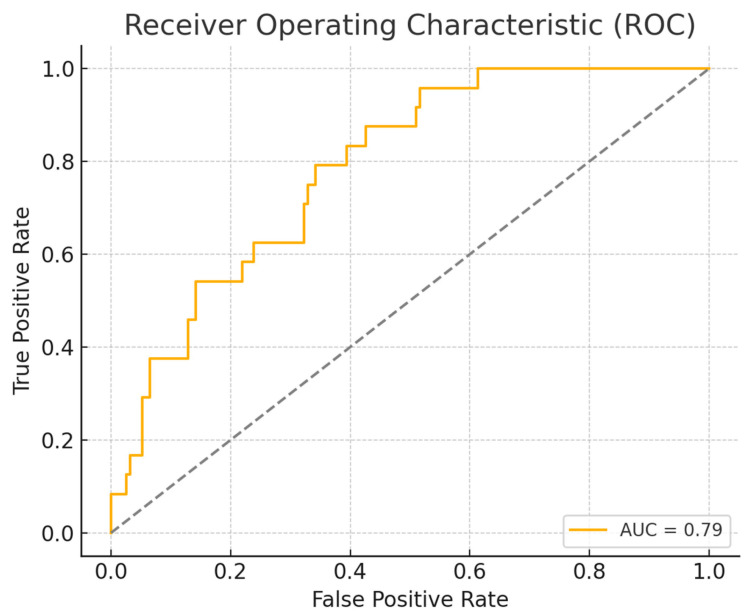
ROC curve for the multivariable logistic regression model. Abbreviations: AUC—area under the curve.

The discriminatory ability of the multivariable logistic regression model was assessed using ROC curve analysis. The model demonstrated good predictive performance, with an AUC of 0.79, indicating a good ability to distinguish between patients who did and did not require blood transfusion (Figure 2). To further evaluate the clinical utility of the model, we calculated additional performance metrics based on the subset of patients with complete preoperative data. Using Youden’s index to identify the optimal classification threshold (0.069), the model achieved a sensitivity of 80.0% and a specificity of 78.8%.

## 4. Discussion

In this retrospective single-center study, we aimed to identify preoperative clinical and laboratory predictors of transfusion requirement in patients undergoing total hip or knee arthroplasty. Recognizing the impact of perioperative blood loss and its management on patient outcomes, we analyzed a wide range of baseline characteristics, with particular focus on sex, age, BMI, hemoglobin, RBC count, INR, APTT, and the use of TXA.

Our findings confirmed that lower preoperative hemoglobin and RBC levels, lack of TXA use, and undergoing THA (compared to TKA) were independently associated with increased transfusion risk. Interestingly, while INR did not reach significance in univariate testing, it emerged as a relevant factor in the multivariable model. This discrepancy likely reflects the effect of covariate adjustment in the multivariable model. Variables such as hemoglobin, RBC count, and TXA use may confound the isolated association of INR with transfusion risk. When these factors are held constant, the independent contribution of INR becomes more apparent. Such findings are not uncommon in multivariable analysis, particularly when interdependencies exist between hematologic and coagulation parameters. While we did not formally test for interactions, the potential modifying effect of other variables on the role of INR warrants further investigation in larger, stratified cohorts. These results support the inclusion of standard hematologic parameters in preoperative risk stratification protocols and highlight the protective role of TXA in blood conservation strategies. However, given the retrospective design, this association should be interpreted cautiously. The observed effect may reflect underlying clinical decisions, unmeasured confounders, or selection bias. Therefore, we cannot infer a direct causal relationship between TXA omission and transfusion risk.

Although transfusion rates may differ between THA and TKA, we chose to analyze both procedures within a single model while adjusting for surgery type, reflecting real-world clinical settings where both procedures follow unified perioperative protocols. This approach has been applied in other studies [17,22], allowing for the identification of shared and independent predictors across arthroplasty types. Importantly, our multivariable analysis confirmed that surgery type (THA vs. TKA) remained an independent predictor of transfusion, thus statistically addressing this heterogeneity. Nonetheless, future research may benefit from stratified models or procedure-specific predictive tools, particularly if perioperative management becomes more differentiated between THA and TKA over time.

Although our study population did not include patients with inherited coagulopathies, the broader concept of individualized hemostatic assessment remains relevant. As highlighted by Badulescu et al. [21], structured perioperative strategies including patient-specific coagulation profiles and tailored transfusion thresholds can optimize outcomes in orthopedic surgery, especially in high-risk populations. These insights support further refinement of preoperative evaluation protocols, even beyond rare bleeding disorders. Presented findings are consistent with those reported by Frisch et al. [22], who conducted a large retrospective analysis of 1573 patients undergoing primary THA and TKA. In their study, transfusion rates were significantly higher in THA (26.6%) compared to TKA (9.27%), which mirrors the trend observed in our cohort. They identified several significant predictors of transfusion, including lower preoperative hemoglobin, female sex, advanced age, lower BMI, elevated creatinine, longer operative time, and greater intraoperative blood loss. Although our dataset did not include intraoperative blood loss or surgical duration, we similarly found that low hemoglobin and undergoing THA were independently associated with increased transfusion risk. Moreover, Frisch et al. reported a higher rate of deep surgical site infections among transfused patients, emphasizing the potential clinical consequences of transfusion. These results underscore the importance of perioperative optimization strategies aimed at minimizing transfusion, such as early anemia correction and judicious fluid management.

Our results also align with the findings of Pempe et al. [17], who retrospectively analyzed 308 patients undergoing primary TKA or THA to identify risk factors for blood loss and RBC transfusion. In their study, preoperative hemoglobin concentration and the use of surgical drains were the most prominent predictors of transfusion, while the role of TXA appeared less pronounced in multivariate analysis. Notably, they demonstrated that transfusion probability was strongly dependent on preoperative hemoglobin levels, with patients in the 9.0–10.0 g/dL range having a 100% transfusion rate. Similarly, in our cohort, lower preoperative hemoglobin was a strong independent predictor of transfusion, and patients who received TXA had significantly lower transfusion rates, supporting its effectiveness in clinical practice. Although our study did not collect data on drain use or mechanical heart valves, the concordant emphasis on preoperative anemia highlights the value of early identification and potential correction as part of a targeted blood management strategy. Furthermore, the concept of individualized transfusion probability proposed by Pempe et al. reinforces the need for personalized perioperative planning in high-risk arthroplasty patients.

The concept of preoperative risk stratification for transfusion is further supported by the work of Hayter et al. [18], who developed a predictive model to identify American Society of Anaesthesiologists I–II patients unlikely to require RBC transfusion following elective THA or TKA. Their model, based on 13 preoperative variables, demonstrated excellent performance (AUC = 0.945), highlighting the feasibility of selecting low-risk patients for streamlined surgical pathways. Although our study did not construct a predictive model per se, we similarly identified key baseline variables, particularly hemoglobin level and the use of TXA, that were independently associated with transfusion requirement. While Hayter et al. focused on safely reducing preoperative testing and resource use in healthy patients, our findings may complement such strategies by identifying higher-risk individuals who may benefit from enhanced perioperative management, such as preoperative anemia correction or more intensive monitoring. Together, these results underscore the clinical value of preoperative data in optimizing resource allocation and improving patient outcomes in major joint arthroplasty.

The economic impact of allogeneic blood transfusions in arthroplasty has been well documented, most notably in the large international study by Blanchette et al. [23], which analyzed over 220,000 hospitalizations in the United States and Belgium. Their findings demonstrated that transfusion services significantly increase healthcare costs, with an average hospitalization cost of $12,718 in the U.S., and substantially lower costs in Belgium ($6526), where the use of blood products was more prevalent. Interestingly, despite shorter hospital stays in the U.S., patients there were 88% less likely to receive an allogeneic transfusion, largely due to differences in perioperative management and pharmacologic strategies such as TXA use. These findings underscore the cost-saving potential of effective blood conservation strategies. In our study, the use of TXA was associated with a markedly reduced transfusion rate, supporting its clinical and economic value. Collectively, this evidence reinforces the importance of identifying high-risk patients preoperatively, not only to reduce transfusion-related complications but also to optimize resource utilization and contain costs in joint replacement surgery.

The importance of appropriate transfusion practices is further emphasized by the findings of Saporito et al. [24], who conducted a large retrospective analysis of nearly 55,000 surgical patients and found that 56% of all RBC transfusions were administered inappropriately based on international guidelines. Inappropriately transfused patients had significantly longer hospital stays and incurred an average of USD 9779 in additional costs compared to non-transfused patients. These findings highlight not only the clinical but also the economic burden of suboptimal transfusion practices. Although our study did not directly assess the appropriateness of transfusions, the identification of preoperative risk factors—particularly low hemoglobin and lack of TXA use—may contribute to reducing unnecessary transfusions through better patient selection and optimization. Moreover, our results support the integration of evidence-based transfusion thresholds and PBM strategies to improve both clinical outcomes and cost-effectiveness in elective orthopedic surgery.

Although our model demonstrated acceptable discriminative performance and identified clinically relevant predictors, we did not construct a formal risk score or nomogram. Such tools can help translate statistical models into point-of-care decision aids by enabling individualized transfusion risk estimation. Developing a validated, user-friendly nomogram based on our predictors such as hemoglobin level, INR, TXA use, and surgery type may improve the practical implementation of preoperative risk stratification protocols in orthopedic surgery. Future studies, particularly those with prospective design and richer intraoperative data, should aim to build and validate such tools.

A major strength of our study is the inclusion of a well-defined, consecutive cohort of patients undergoing primary THA and TKA, reflecting real-world clinical practice. The single-center design ensured consistency in surgical technique, perioperative protocols, and transfusion thresholds. Furthermore, the evaluation of TXA use allowed us to highlight its significant role in transfusion risk reduction. By using multivariable analysis, we were able to identify independent predictors while adjusting for potential confounders, thereby enhancing the robustness of our findings. Taken together, these factors strengthen the clinical relevance and applicability of our results to perioperative risk assessment and blood management planning in arthroplasty patients. Identifying patients at higher risk of transfusion allows for early interventions, including anemia correction, TXA administration, and informed patient counseling.

This study has several limitations. First, its retrospective and single-center design may limit generalizability to other populations or healthcare settings. Additionally, institutional transfusion thresholds, documentation practices, and perioperative protocols, particularly regarding TXA administration, may differ across centers. These differences could influence transfusion rates and the predictive strength of certain variables, underscoring the need for external validation in diverse clinical settings. Second, although we analyzed a relatively large sample, the dataset may still be underpowered to detect associations with less frequent risk factors. Third, transfusion decisions were partially based on clinical judgment, which could introduce variability despite standardized institutional thresholds. Fourth, we did not account for intraoperative blood loss volume and duration of surgery, which are known contributors to transfusion risk. Lastly, the potential influence of unmeasured confounders, such as nutritional status, undiagnosed coagulation disorders, estimated blood loss, operative time, and use of surgical drains cannot be fully excluded.

## 5. Conclusions

This retrospective study identified several preoperative predictors of transfusion requirement in patients undergoing TKA or THA. Lower hemoglobin and RBC levels, higher INR, absence of TXA use, and undergoing THA rather than TKA were independently associated with increased transfusion risk. While these findings suggest that routine preoperative hematologic and coagulation assessments as well as consideration of TXA use may support more effective perioperative blood management, the proposed model requires external validation before it can be applied in clinical practice. Future prospective studies are warranted to confirm these associations and assess the model’s utility in improving transfusion-related outcomes in major joint arthroplasty.

## Figures and Tables

**Table 1:** Patients’ characteristics.

Variable	(*n* = 742)
Age (years)	65.8 ± 9.9
BMI (kg/m^2^)	30.1 ± 4.6
Women (%)	61.1%
Surgery type	
TKA	45.5%
THA	54.5%
HGB before surgery (g/dL)	13.9 ± 4.2
HGB after surgery (g/dL)	11.3 ± 1.6
RBC before surgery (million/μL)	4.5 ± 0.8
RBC after surgery (million/μL)	3.7 ± 0.5
INR	1.0 ± 0.2
APTT (s)	28.0 ± 3.6
TXA use prior to surgery (%)	46.9%
Transfusion needs (%)	12.0%

Results are presented as mean ± SD or as percentages. Abbreviations: APTT—activated partial thromboplastin time; BMI—body mass index; HGB—hemoglobin; INR—international normalized ratio; RBC—red blood cell count; SD—standard deviation; THA—total hip arthroplasty; TKA—total knee arthroplasty; TXA—tranexamic acid.

**Table 2:** Comparison of patients with and without transfusion need.

Variable	Transfusion (*n* = 89)	No Transfusion (*n* = 653)	*p*-Value
Age (years)	69.1 ± 11.1	65.4 ± 9.6	0.0040
BMI (kg/m^2^)	28.9 ± 5.0	30.2 ± 4.5	0.0169
Women (%)	75.3%	59.2%	0.0050
Surgery type			
Total knee arthroplasty	37.1%	46.7%	0.1111
Total hip arthroplasty	62.9%	53.3%	0.1111
HGB before surgery (g/dL)	12.4 ± 1.8	14.2 ± 4.4	<0.0001
HGB after surgery (g/dL)	8.4 ± 0.6	11.7 ± 1.3	<0.0001
RBC before surgery (million/μL)	4.3 ± 1.1	4.6 ± 0.7	0.0221
RBC after surgery (million/μL)	2.8 ± 0.3	3.8 ± 0.4	<0.0001
INR	1.0 ± 0.1	1.0 ± 0.2	0.2711
APTT (s)	27.8 ± 2.7	28.0 ± 3.8	0.4708
TXA use prior to surgery (%)	18.0%	50.8%	<0.0001
Transfusion volume (units)	2.1 ± 0.8	-	-

Results are presented as mean ± SD or as percentages. Abbreviations: APTT—activated partial thromboplastin time; BMI—body mass index; HGB—hemoglobin; INR—international normalized ratio; RBC—red blood cell count; SD—standard deviation; THA—total hip arthroplasty; TKA—total knee arthroplasty; TXA—tranexamic acid.

**Table 3:** Univariable logistic regression analysis of factors associated with blood transfusion.

Variable	Coefficient	OR (95% CI)	*p*-Value
Age (years)	0.04	1.04 (1.02–1.07)	0.0018
BMI (kg/m^2^)	−0.07	0.93 (0.88–0.98)	0.0097
HGB before surgery (g/dL)	−0.64	0.53 (0.44–0.63)	<0.0001
RBC before surgery (million/μL)	−0.82	0.44 (0.26–0.76)	0.0030
INR	−1.12	0.33 (0.01–11.61)	0.5383
APTT (s)	−0.02	0.98 (0.92–1.05)	0.6236
Male sex	−0.70	0.50 (0.29–0.84)	0.0096
Surgery type tTKA	−0.48	0.62 (0.38–1.00)	0.0496
TXA use prior to surgery	−1.53	0.22 (0.11–0.41)	<0.0001

Abbreviations: APTT—activated partial thromboplastin time; BMI—body mass index; CI—confidence interval; HGB—hemoglobin; INR—international normalized ratio; OR—odds ratio; RBC—red blood cell count; THA—total hip arthroplasty; TKA—total knee arthroplasty; TXA—tranexamic acid.

**Table 4:** Multivariable logistic regression model of independent predictors of blood transfusion.

Variable	Coefficient	OR (95% CI)	*p*-Value
Age (years)	0.02	1.02 (0.99–1.05)	0.1552
BMI (kg/m^2^)	−0.04	0.96 (0.90–1.03)	0.2331
Male sex	−0.31	0.74 (0.38–1.42)	0.3604
HGB before surgery (g/dL)	−0.79	0.45 (0.37–0.56)	<0.0001
RBC before surgery (million/μL)	−0.53	0.59 (0.45–0.78)	0.0003
INR	−4.71	0.01 (0.00–0.95)	0.0475
APTT (s)	−0.0	1.0 (0.92–1.08)	0.9305
Surgery type TKA	−0.88	0.42 (0.23–0.75)	0.0034
TXA use prior to surgery	−2.15	0.12 (0.05–0.25)	<0.0001

Abbreviations: APTT—activated partial thromboplastin time; BMI—body mass index; CI—confidence interval; HGB—hemoglobin; INR—international normalized ratio; OR—odds ratio; RBC—red blood cell count; THA—total hip arthroplasty; TKA—total knee arthroplasty; TXA—tranexamic acid.

## Data Availability

The data that support the findings of this study are available on request from the corresponding author. The data are not publicly available due to their containing information that could compromise the privacy of research participants.
